# miRNA Dysregulation in the Development of Non-Alcoholic Fatty Liver Disease and the Related Disorders Type 2 Diabetes Mellitus and Cardiovascular Disease

**DOI:** 10.3389/fmed.2020.527059

**Published:** 2020-09-22

**Authors:** Andrea R. López-Pastor, Jorge Infante-Menéndez, Óscar Escribano, Almudena Gómez-Hernández

**Affiliations:** ^1^Biochemistry and Molecular Biology Department, School of Pharmacy, Complutense University of Madrid, Madrid, Spain; ^2^Centro de Investigación Biomédica en Red (CIBER) of Diabetes and Associated Metabolic Diseases, Instituto de Salud Carlos III, Madrid, Spain; ^3^Instituto de Investigación Sanitaria Hospital Clínico San Carlos, Instituto de Salud Carlos III, Madrid, Spain

**Keywords:** non-alcoholic fatty liver disease, microRNAs, type 2 diabetes mellitus, cardiovascular disease, obesity

## Abstract

According to the World Health Organization, the continuing surge in obesity pandemic creates a substantial increase in incidences of metabolic disorders, such as non-alcoholic fatty liver disease (NAFLD), type 2 diabetes mellitus, and cardiovascular disease. MicroRNAs (miRNAs) belong to an evolutionarily conserved class of short (20–22 nucleotides in length) and single-stranded non-coding RNAs. In mammals, miRNAs function as critical post-transcriptional negative regulators involved not only in many biological processes but also in the development of many diseases such as NAFLD and comorbidities. More recently, it has been described that cells can secrete miRNAs in extracellular vesicles, transported by body fluids, and uptaken by other tissues regulating gene expression. Therefore, this could be a mechanism of signaling involved not only in physiological pathways but also in the development of diseases. The association of some miRNA expression profiles with certain disorders has made them very interesting molecules for diagnosis, prognosis, and disease management. The finding of specific miRNA signatures to diagnose NAFLD and related diseases could anticipate the risk of development of related complications and, actually, it is the driving force of present health strategies worldwide. In this review, we have included latest advances in knowledge about the miRNAs involved in the development of NAFLD and related diseases and examined how this knowledge could be used to identify new non-invasive biomarkers and new pharmacological interventions.

## Introduction

Nowadays, due to the high prevalence of non-alcoholic fatty liver disease (NAFLD) worldwide, it is considered as the most common hepatic disorder in western countries, with a prevalence of 15–30% (1–4). This condition represents the hepatic manifestation of metabolic syndrome since it is strongly associated with obesity, insulin resistance (IR), hypertension, and dyslipidemia (1). More precisely, NAFLD is defined as the presence of steatosis in >5% of hepatocytes, determined by liver histology, without any evidence of other factors related to the development of fatty liver, such as drug and alcohol abuse, viral hepatitis, or autoimmunity (5, 6).

Obesity and type 2 diabetes mellitus (T2DM) are common and well-established risk factors for NAFLD since increased body mass index (BMI) and visceral adiposity are critically involved in NAFLD development. Although it is true that NAFLD patients do not necessarily develop T2DM, the association between both diseases has been thoroughly described. This significant association has been attributed to IR, the main cause for lipid overflow to the liver (7, 8). Moreover, NAFLD is associated with increased mortality in relation to cardiovascular disease (CVD), neoplastic processes, and hepatic failure (9).

The earliest stage is characterized by benign steatosis (fatty liver) (9), which can progress to non-alcoholic steatohepatitis (NASH), a potentially severe condition from which 10–25% of patients finally develop cirrhosis. This stage is defined by lobular and portal inflammation, hepatocyte ballooning, and variable degrees of fibrosis, cirrhosis, and, ultimately, hepatocellular carcinoma (HCC) (7). Due to the high prevalence mentioned above, NASH is predicted to surpass hepatitis C as the leading cause of liver transplantation in the near future (2–4). Several factors influence the progression from steatosis to NASH, including lipotoxicity, oxidative stress, and activation of the immune system, although many others may be involved and remain unclear (10).

Bearing in mind all of these, identification of patients who might be at an increased risk of adverse outcomes is critical; therefore, the increase in knowledge of serum biomarkers could be of great interest in order to allow an early diagnosis. The treatment of NASH patients should be a priority, especially for those who develop concomitant fibrosis since they are prone to have adverse outcomes. In that sense, treatment goals depend on the severity of the disease due to the likelihood of progression and the comorbidities that each patient might have. Lifestyle-focused treatment strategies are essential regardless of the disease stage, keeping in mind that other complementary therapies could be beneficial according to the patient circumstances (10).

Even though certain genetic risk factors are linked to steatohepatitis and fibrosis degree, they are fairly infrequent. As a result, they are not currently suitable to identify appropriate individuals for therapy since they do not account for a large-enough proportion of the variability in the phenotype of the disease (11–13).

In the recent years, there has been great interest in the function and the usefulness of microRNAs (miRNAs) as clinical tools. The miRNAs are small (20–22 nucleotides), non-coding, highly conserved endogenous RNAs that regulate gene expression at the post-transcriptional level. It has been demonstrated that the expression of the majority of mammal genes is regulated by miRNAs; therefore, these molecules have crucial roles in numerous physiological processes such as cell growth, embryonic development, and apoptosis. It has been demonstrated that an altered expression of some miRNAs is enough to promote the development and the progression of pathophysiological processes including NAFLD and T2DM (14, 15). This review summarizes the data published regarding the role of miRNAs in the development of NAFLD and associated metabolic diseases such as T2DM and CVD, exploring their role in diagnosis and as potential targets for treatment. Therefore, further characterization of the mechanisms that regulate the expression and the function of these small non-coding RNA molecules is essential to develop new therapeutic strategies. Furthermore, not only do these miRNAs regulate gene expression in the tissues in which they are expressed but also they are secreted to the bloodstream in extracellular vesicles to regulate gene expression in different tissues. As it has been described that the dysregulation of some miRNA expression is involved in the development of NAFLD and the associated complications, much effort is essential to increase the knowledge regarding the underlying mechanisms, which would allow an increase in the therapeutic potential for this group of diseases.

## Biology of miRNAs

As mentioned before, miRNAs belong to an evolutionarily conserved class of short (20–22 nucleotides in length) and single-stranded non-coding RNAs. In mammals, miRNAs take part in gene expression regulation by its repression at the post-transcriptional level. Consequently, they are involved in a huge number of biological processes including cell growth, tissue differentiation, cell proliferation, embryonic development, and apoptosis (16, 17). Such regulation can be carried out owing to their ability to target the 3′ untranslated region of a gene mRNA, resulting in translational repression, mRNA degradation, or mRNA cleavage, based on the complementarity between the miRNA and its target (18). It is known that ~1–4% of the protein-encoding loci in the human genome also encode one or more miRNAs and a single miRNA can regulate as many as 200 cognate mRNAs.

miRNAs are synthesized by RNA polymerase II in the nucleus and, immediately, they are cleaved into pre-miRNAs by the endonuclease Drosha. After processing, the Ran-GTPase Exportin-5 exports pre-miRNAs into the cytoplasm, allowing Dicer to process them into 20–22-nucleotide-long mature miRNAs. Then, miRNAs are loaded into Argonaute-2 of the RNA-induced silencing complex in order to direct the post-transcriptional repression of target mRNAs (16).

Aberrant miRNA expression can foster the onset and the progression of a range of pathophysiological processes (17). Indeed the literature has recently highlighted the role of circulating miRNAs as potential diagnostic and prognostic biomarkers (18). Several studies have evaluated their levels in different chronic diseases including chronic liver disease (19–26). For instance, patients with hepatitis B virus-related hepatocellular carcinoma and/or chronic type B hepatitis showed increased levels of serum miR-21, miR-122, and miR-223, pointing toward their potential use as diagnostic biomarkers for liver injury and potential target for treatment (27). Moreover, between all the miRNAs described in the literature, only miR-133a/b, miR-208a/b, and miR-499 were well-established as possible biomarkers. Recently, within liver damage diseases, other authors have highlighted the importance of miR-122, miR-29a, and miR-34a as relevant biomarkers for the diagnosis of this spectrum of diseases (28–30).

The unexpected stability of miRNAs in the circulation points toward a signaling pathway in which miRNAs are selectively secreted by one cell and taken up by a distant target cell to regulate gene expression. This intriguing idea of circulating miRNAs regulating distant cell-to-cell communication has been intensively investigated. In this sense, a recent and elegant paper demonstrates that adipose-derived circulating miRNAs regulate gene expression in other tissues such as the liver (31). However, the mechanisms involved in these processes are poorly understood. Huge effort is needed in order to know the specific targets of miRNAs in cells and the molecular mechanisms playing a role in the development and the progression of NAFLD and related outcomes.

## miRNAs Involved in Nafld

Thanks to the efficient distribution and accumulation of exogenously administered small RNAs in the liver, it is likely that liver diseases will have miRNA-based therapies in the near future (32).

**Figure 1:**
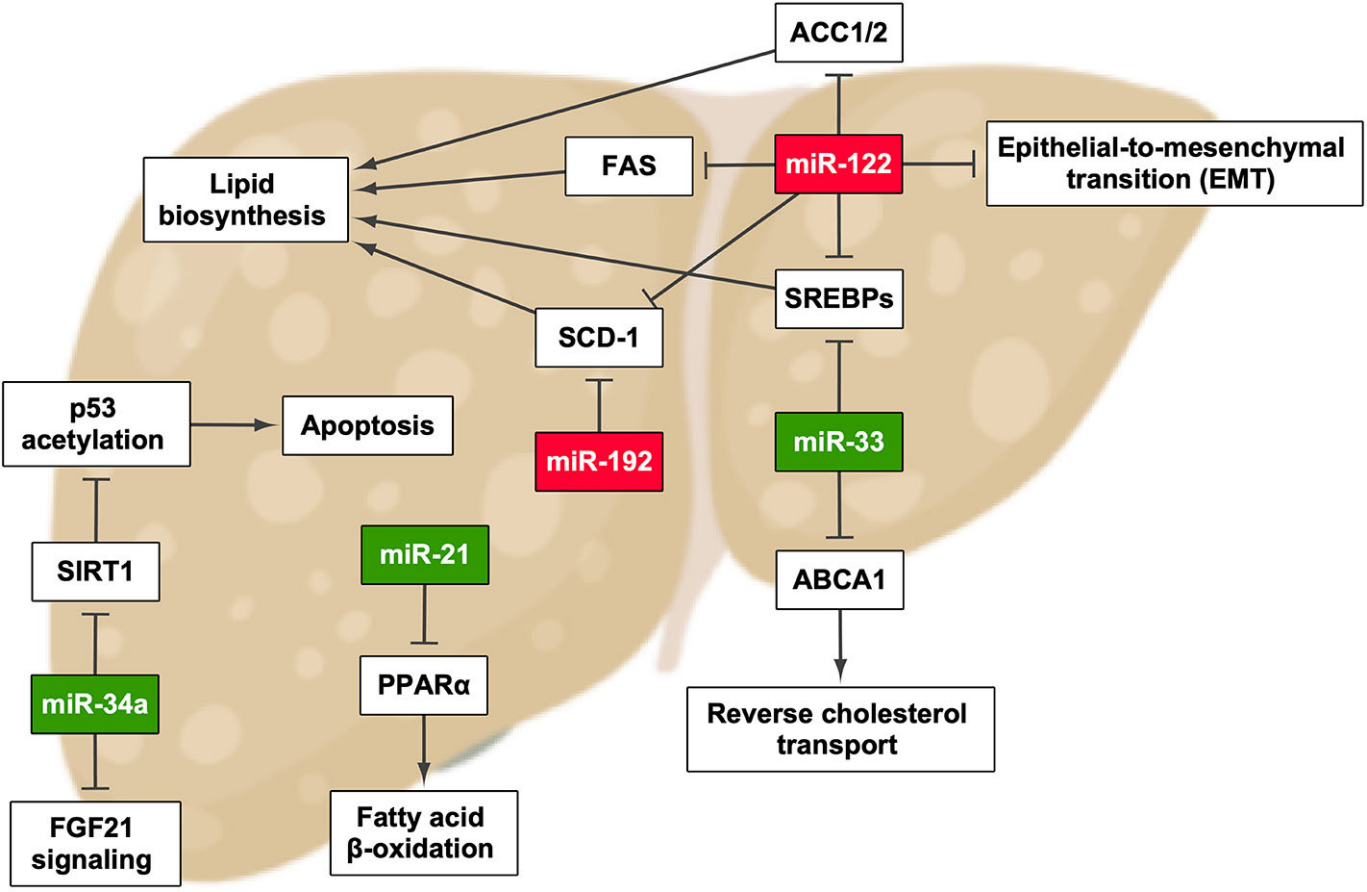
miRNAs that have been described to promote the development and/or progression of non-alcoholic fatty liver disease and their effect in pathways involved in this process. Green highlights indicate the upregulation of miRNA, whereas red highlights indicate its downregulation. ACC1/2, acetyl-CoA carboxylase 1/2; FAS, fatty acid synthase. Created with BioRender.com.

Many studies have described that aberrant miRNA expression is a main feature of liver diseases, including NAFLD, viral hepatitis, and HCC (Figure 1 and Table 1). Moreover, it has been demonstrated that changes in miRNA expression are well-correlated with NAFLD progression (26, 33, 34). As mentioned before, circulating miRNAs are quite stable in body fluids, and therefore it would be reasonable to think that changes in the circulating miRNA pattern could represent a real-time signal of how NAFLD evolves, reflecting variations at the histological and the molecular levels (34). Hence, establishing the exact miRNA signature in NAFLD is fundamental to unravel the development mechanism and to enable the early diagnosis and severity evaluation of this disease.

**Table 1:** Summary of miRNAs aberrantly expressed in non-alcoholic fatty liver disease (NAFLD), type 2 diabetes mellitus (T2DM), and cardiovascular disease (CVD).

**miRNA**	**Expression changes**	**Organism**	**Sample**	**References**
**NAFLD**				
miR-21	↑	Human	Serum	(52–54)
miR-33	↑	HepG2 and Huh7 cells, Mice, rat, and human	Liver	(55–57)
miR-34a	↑	Mice, rat, and human	Serum	(43–48)
miR-122	↓	Primary cultured murine Hepatocytes, mice, and human	Liver	(29, 36–40)
miR-125a-5p	↓	Mice	Liver	(35)
miR-155	↓	Mice	Liver	(87)
miR-182	↑	Mice	Liver	(35)
miR-192	↓	Rat and human	Liver	(29, 36, 37, 39–41, 44, 49–51)
miR-223	↑	Human	Serum	(27)
miR-335	↑	Mice	Liver	(42)
miR-340-5p	↓	Mice	Liver	(35)
miR-484	↑	Mice	Liver	(35)
miR-574-3p	↑	Mice	Liver	(35)
miR-720	↓	Mice	Liver	(35)
**T2DM**				
let-7	↑	C2C12 cells and mice	Liver and skeletal muscle	(74, 75)
miR-1	↓	Mice and human	Skeletal muscle	(72, 73)
miR-7a	↑	MIN6 cells, mice, and human	Pancreas	(66, 67)
miR-17	↑	Human	Serum	(39)
miR-20a/b	↑	Human	Serum	(39)
miR-29	↑	3T3-L1 and Huh7 cells, mice, and rat	Liver, adipose tissue, and skeletal muscle	(69–71)
miR-30d	↑	MIN6 cells	Pancreas	(63)
miR-107	↑	MIN6 cells	Pancreas	(63)
miR-124a	↑	MIN6 cells	Pancreas	(63)
miR-133a	↓	Mice and human	Skeletal muscle	(72, 73)
miR-145	↓	Human	Circulating monocytes	(89–92)
miR-200	↑	MIN6 cells and mice	Pancreas	(68)
miR-296	↓	MIN6 cells	Pancreas	(63)
miR-375	?	MIN6 cells and mice	Pancreas	(64, 65)
miR-484	↓	MIN6 cells	Pancreas	(63)
miR-690	↓	MIN6 cells	Pancreas	(63)
**CVD**				
let-7	↓	Human	Atherosclerotic plaques and serum	(93, 96)
miR-1	↓	Human	Atherosclerotic plaques	(96)
miR-15a-5p	↑	Human	Serum	(95)
miR-16-5p	↑	Human	Serum	(95)
miR-17	↓	Human	Serum	(92)
miR-21	↑	Human	Atherosclerotic plaques	(96)
miR-22	↓	Human	Atherosclerotic plaques	(96)
miR-34a	↑	Human	Serum	(51)
miR-92a	↓	Human	Serum	(92)
	↑	Human	Atherosclerotic plaques	(96)
miR-93a-5p	↑	Human	Serum	(95)
miR-99a	↑	Human	Atherosclerotic plaques	(96)
miR-122	?	Human	Serum	(76, 82, 83)
miR-126	↓	Human	Serum	(92)
miR-132	↓	Human	Serum	(86)
miR-133a	↑	Human	Serum	(92)
miR-143	↑	Human	Serum	(86)
miR-145	↓	Mice and human	Serum, murine aorta, and human carotid	(89–92)
miR-146a-5p	↓	Human	Serum	(95)
miR-155	↑	Mice	Aorta	(87)
	↓	Human	Serum	(88, 92, 93)
miR-208	↑	Human	Serum	(92)
miR-212	?	Human	Serum	(94)
miR-372	?	Human	Serum	(94)
miR-454	?	Human	Serum	(94)
miR-744	?	Human	Serum	(94)

A study explored the dynamics of miRNA expression during NAFLD progression in mice fed a high-fat diet (HFD) for a long term. miR-125a-5p and miR-182 were found to be altered in the early stages of NAFLD, and miR-340-5p, miR-484, miR-574-3p, and miR-720 were related to liver damage and tumor development. miRNA expression profiles were also different depending on the NAFLD stage, suggesting that its progression involves diverse miRNAs (35).

### miR-122

miR-122 is the main miRNA in the liver and is involved in many biological processes. A mouse model of liver damage, by feeding it with a methyl-deficient diet, showed a decreased expression of miR-122 (36). The same was found in NASH patients but not in normal subjects or simple steatosis patients (29). In addition, mice with *Mir122a* deletion induced steatosis that led to NASH, fibrosis, and HCC, suggesting an essential role of this miRNA in NAFLD initiation and progression (36). In that report, the authors showed that miR-122 is essential in processes such as fatty acid, triglyceride, and cholesterol metabolism and also in terminal differentiation of hepatocytes by regulating its targets, including *FASN, ACC, SCD1*, and *SREBPs*, among others (36–40). miR-122 knockout mice showed decreased lower serum cholesterol and triglyceride levels but increased cholesterol and triglyceride content within the liver. The alterations in very-low-density lipoprotein assembly and secretion are responsible for changes in cholesterol and triglyceride levels in the serum and the liver in a miR-122-dependent manner (36). However, another study found that inhibiting miR-122 with antisense oligonucleotides in HFD and normal-diet mice decreased the expression of lipogenic genes and increased hepatic fatty acid oxidation, ameliorating hepatocyte steatosis (40). This controversy has been observed in several studies and could be due to the use of distinct models and inhibition approaches. It is of great necessity to further study deeper on how miR-122-dependent and miR-122-independent pathways can regulate hepatic lipid metabolism *in vivo*.

Moreover, miR-122 could also be important in NAFLD diagnosis since it has been reported that miR-122, along with miR-17 and miR-20a/b, plasma levels were increased in T2DM patients with NAFLD compared to those diabetic patients without NAFLD complication (39).

In addition, miR-122 plays a role in the fibrogenic and the carcinogenic signaling pathways of NAFLD. Reduced miR-122 expression resulted in the activation of MEKK-3, vimentin, and hypoxia-inducible factor-1α, factors involved in epithelial to mesenchymal transition, a fundamental process related to chronic inflammation, fibrosis, and metastasis (41). Furthermore, it has also been described that the loss of miR-122 fosters liver fibrosis and promotes the activation of oncogenic miRNAs (37). All these results demonstrate that miR-122 could be considered as an important biomarker in NAFLD diagnosis and staging.

### miR-335

A different work reported that, in mice, miR-335 could be a biomarker for steatosis since higher levels of this miRNA were well-correlated with hepatic steatosis in genetically obese mice (*ob/ob* and *db/db*) (42). Although the quantity and the quality of reports regarding the role of miRNAs in NAFLD are increasing every week, much effort is needed in order to clarify the multifaceted roles of these molecules.

### miR-34a

Another miRNA that seems to be important in NAFLD development is miR-34a. For instance, one study performed in 34 patients showed that this miRNA was only detected in the serum of NAFLD/NASH patients, but not in healthy subjects, which has been also supported by Liu et al. (43, 44). Indeed this aberrant increase negatively impacts the signaling of the fibroblast growth factors 19 and 21, and it is positively correlated with BMI in obese patients (45, 46). Moreover, miR-34a expression is triggered by lipids, and as sirtuin 1 has been identified as one of its targets, this miRNA seems to have a role in aggravating the manifestations of NAFLD and NASH, mainly through hepatocyte apoptosis induction by increasing p53 acetylation (47, 48). This evidence suggests that miR-34a silencing might be supposed as a new therapeutic strategy for NAFLD treatment.

### miR-192

Among other miRNAs involved in NAFLD, miR-192 stands out due to its implication in lipid synthesis through targeting stearoyl-CoA desaturase 1; therefore, its upregulation would be an approach to treat the disease (49, 50). It has been also described that, like miR-122, miR-192 expression is lower in the liver of NASH patients in contrast with NAFLD patients (29, 44, 51).

### miR-21

Becker et al. assessed the serum profiles of two cohorts consisting of 137 NAFLD/NASH patients and 61 healthy controls, and the results showed an increase in circulating miR-21 levels in patients suffering from NASH compared to healthy controls and NAFLD patients (52). The authors mentioned that it might be due to increased fibrosis in NASH patients; thus, miR-21 plays a pathogenic role by targeting peroxisome proliferator-activated receptor alpha, an important factor for the progression of the disease (53, 54).

### miR-33

The NAFLD patients also show upregulated miR-33 expression in the liver (55), and it has been found to be a regulator of lipid metabolism and transport and insulin signaling pathways, its main targets being sterol regulatory element-binding proteins (SREBPs) and ATP binding cassette subfamily A member 1 (56–58). Moreover, it has been demonstrated that miR-33 inhibition attenuates atherosclerosis progression (57, 58).

## miRNAs Involved in Type 2 Diabetes Mellitus

**Figure 2:**
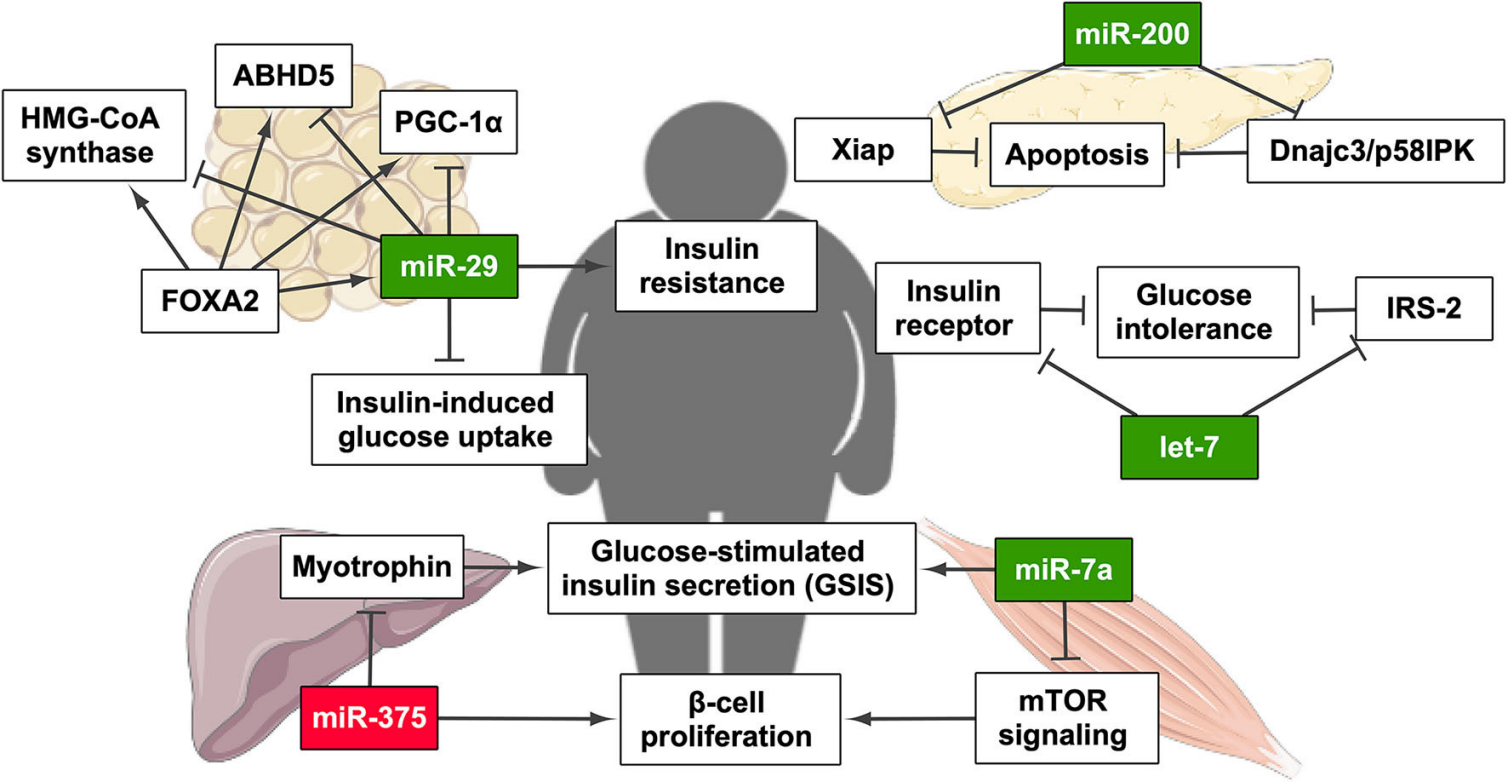
miRNAs related to the establishment and/or progression of type 2 diabetes mellitus (T2DM) and their regulatory effect on T2DM-related pathways. Green highlights indicate the upregulation of miRNA, whereas red highlights indicate its downregulation. PGC-1α, PPARγ coactivator 1α. This figure has been edited from Servier Medical Art. Servier is licensed under a Creative Commons Attribution 3.0 Unported License. Created with BioRender.com.

As has been mentioned above, in the same way that the prevalence of NAFLD in obese patients can exceed 90% (7, 10, 59), patients that suffer from T2DM are at a high risk of developing NAFLD. Classically, high plasma glucose levels are present, owing to metabolic dysregulations that lead to impaired fasting glucose, a pre-diabetic state that might progress to T2DM, characterized by IR and reduced insulin secretion from pancreatic β cells (60). Blood glucose levels exert a real-time regulatory effect on many pancreatic β cells genes, allowing accurate insulin expression and secretion (61). The physiological release, the transcription and mRNA stability, translation, and processing of insulin are all regulated by glucose concentrations in β cells. Moreover, the relative quantity of miRNA transcripts is responsive to variable glucose concentrations (62). The exposure to long-term high-glucose conditions significantly modifies the expression of a large number of miRNAs in a cultured pancreatic β cell line (MIN6). The expression of miR-124a, miR-107, and miR-30d increased, whereas miR-296, miR-484, and miR-690 expression descended under prolonged high glucose conditions. However, many other miRNAs have been involved in several subcellular events essential for glucose-stimulated insulin secretion (GSIS) (63). In this review, we have included the most relevant miRNAs involved in T2DM (Figure 2 and Table 1).

### miR-375

One of the first miRNAs specific of β cells was miR-375, a highly expressed miRNA in pancreatic islets in humans and mice involved in insulin secretion and glucose homeostasis. This study demonstrated that the knockdown of miR-375 increases GSIS in murine pancreatic β cell line (MIN6) and primary β cells (64). Next, the same laboratory result described that the deletion of miR-375 induced a marked decrease in β cell number, provoking a severe diabetic state. Conversely, miR-375 is increased in the pancreatic islets of *ob/ob* mice, which is consistent with the characteristic compensatory pancreatic hyperplasia of the prediabetic stage (65). With these results, the authors concluded that miR-375 is key for physiological glucose metabolism, β cell proliferation, and turnover (64, 65).

### miR-7a and miR-200

Another β-cell-specific miRNA is miR-7a, which has been shown as a negative regulator of β cell proliferation by regulating the protein mammalian target of rapamycin (mTOR) signaling pathway. Wang et al. showed that the decrease of miR-7a activity resulted in increased mTOR signaling and β cell replication in murine pancreatic islets, implying that miR-7a could represent a potential therapeutic target for T2DM treatment (66). miR-7a not only regulates β-cell proliferation but also its functionality since it was found to inhibit GSIS. Moreover, the increased expression of miR-7a in β cells in mice induced diabetes. In contrast, these authors described that both obese and diabetic mouse models and islets from obese and moderately diabetic patients suffered from a decrease in β cell miR-7a expression. All these data suggest that the levels of miR-7a vary depending on the stage of T2DM development, being decreased when β cell proliferation is needed as it occurs in the first stages of insulin resistance compensation (67). In the same way as miR-7a, another remarkable family of miRNAs is miR-200. These miRNAs are highly expressed in the pancreatic islets of the diabetic *db/db* mouse model. Overexpression of miR-200 in mouse β cells induced severe apoptosis that prompted to T2DM under stressed conditions by inhibiting Dnajc3/p58IPK and the caspase inhibitor Xiap. Indeed β cell apoptosis and T2DM pathogenesis are controlled by miR-200 family members (68). Many other miRNAs have been shown to regulate insulin expression and secretion.

### miR-29

In addition to its role in β cell function and development, it has been widely described that miRNAs are also involved in IR in target tissues. For instance, miR-29 expression is enhanced by hyperinsulinemia and/or hyperglycemia in adipocyte-derived 3T3-L1 cells (69). Moreover, in the rat model of T2DM Goto–Kakizaki, the expression of this miRNA was increased in the liver, adipose tissue, and skeletal muscle. In the same report, the authors demonstrated that the overexpression of miR-29 in 3T3-L1 cells diminishes insulin-induced glucose uptake (70). Finally, it has been demonstrated that miR-29 expression is partially regulated by Forkhead Box A2 (FOXA2), and miR-29 also modifies FOXA2-mediated regulation genes including *PPARGC1A, HMGCS2*, and *ABHD5*, key players in lipid metabolism (71).

### miR-1 and miR-133

Some other miRNAs involved in insulin resistance are miR-1 and miR-133a. These miRNAs are specifically expressed in the muscle, and their expression is regulated by insulin through SREBP1c and myocyte enhancer factor 2C (MEF2C) (72). Moreover, insulin-induced SREBP1c activation in human skeletal muscle triggered the subsequent downregulation of miR-1 and miR-133 through the inhibition of MEF2C. Consequently, insulin is unable to regulate miR-1 and miR-133a in the skeletal muscle in T2DM, perhaps due to the altered activation of SREBP1c (72, 73).

### Let-7

Furthermore, the let-7 family of miRNAs is also involved in global glucose homeostasis since they regulate the expression of *INSR* and *IRS2* genes, among others (74, 75). Frost and Olson showed that the suppression of the let-7 family ameliorated glucose intolerance in a mouse model of diet-induced obesity by enhancing insulin signaling in the muscle and the liver (74). Another group demonstrated that let-7g overexpression produces glucose intolerance; however, insulin resistance changes are not detected. By repressing likewise the activity of the let-7 family, thanks to the overexpression of their let-7 negative regulators, LIN28 isoforms, glucose tolerance, and insulin sensitivity were improved (75). Therefore, this miRNA family supposes a new therapeutic target for T2DM treatment.

Despite the increasing amount of reports describing the role of specific miRNAs in T2DM, not much is known about *in vivo* situations (76). In consequence, it is of great interest to validate these situations in animal models in pathophysiological conditions.

## miRNAs Involved in Cardiovascular Disease

**Figure 3:**
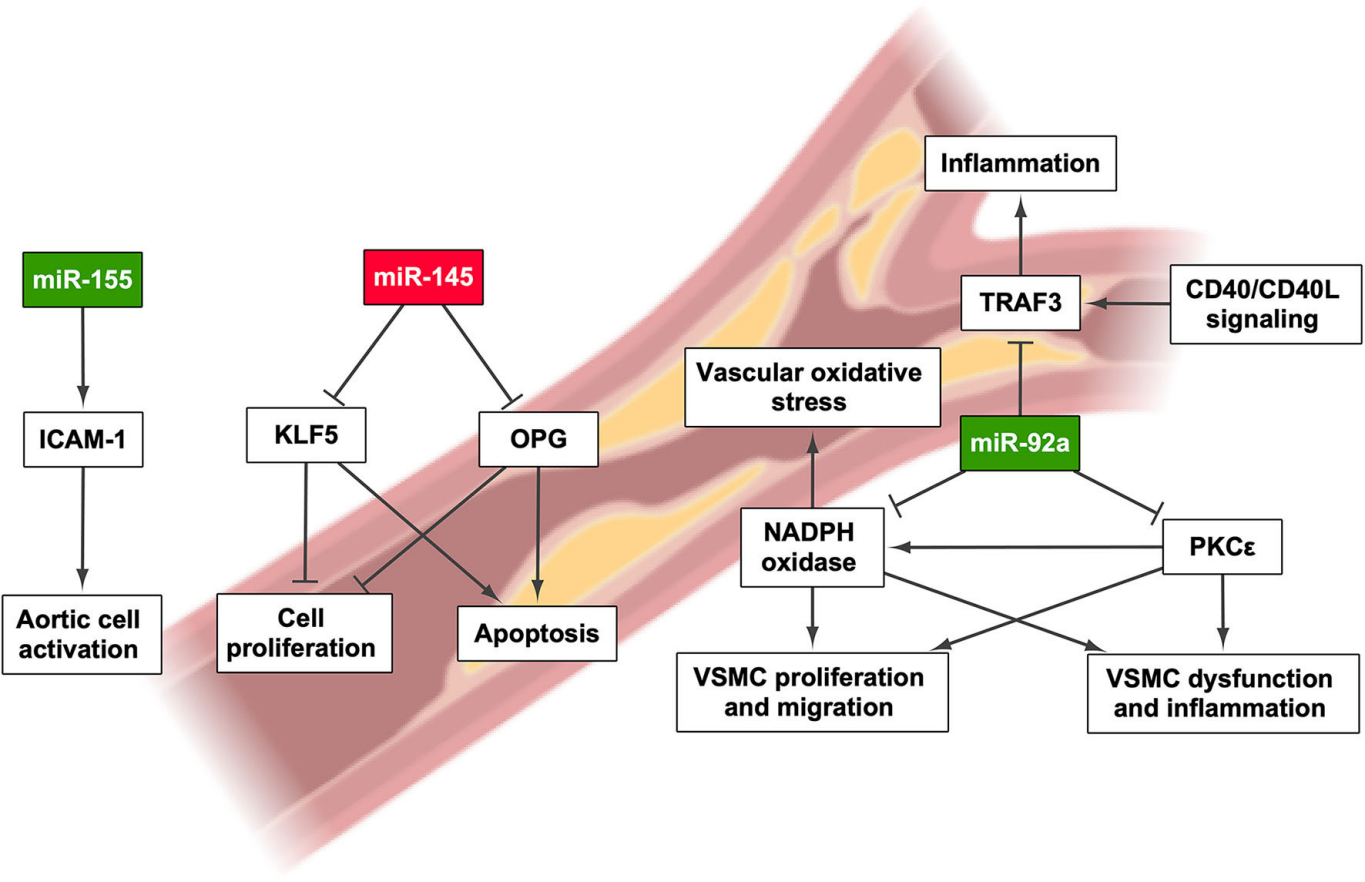
miRNAs involved in the onset and/or progression of cardiovascular disease and their effect on pathways that regulate their development. Green highlights indicate the upregulation of miRNA, whereas red highlights indicate its downregulation. Created with BioRender.com.

Despite the fact that the subjacent pathogenic mechanism linking NAFLD and CVD is still to be determined, several studies have found that NAFLD increases the chances of suffering CVD (77–79). Therefore, NAFLD may cause chronic systemic inflammation, leading to the development of subclinical atherosclerosis and, eventually, CVD (79). The main links between NAFLD and CVD seem to be obesity, fat accumulation in the liver, and secretion of hepatic proteins, including hepatokines, proprotein convertase subtilisin/kexin type 9, and coagulation factors (80). In fact, miRNA secretion from the liver might play an important role in CVD development, especially in relation to CAD, as has been mentioned throughout this review (81) (Figure 3 and Table 1).

### miR-122

Aside from its role in the development of liver diseases, miR-122 also stands out due to its involvement in CVD since it is able to regulate lipid metabolism genes. Nevertheless, the experimental results obtained in clinical studies related to vascular complications are sparse. miR-122 levels are elevated in patients with hyperlipidemia and associated with CAD (82) and acute coronary syndrome (76). Moreover, how statins might reduce the levels of miR-122 in circulation has been described (83). However, other studies have reported negative associations between miR-122 and cardiovascular outcomes (84, 85).

### miR-34a, miR-132, and miR-143

As has been previously described, miR-34a is a potential target for treating NAFLD, but it has been also analyzed in CAD patients, either with or without NAFLD. miR-34a expression is upregulated in patients with CAD, and this is aggravated when the subjects also suffer from NAFLD (51). On the other hand, Mehta et al. described that patients with NAFLD and CAD had a decrease in serum miR-132 levels and an increase in miR-143 expression; thus, they could be used as CAD markers (86).

### miR-155 and miR-145

Another miRNA that seems to be involved in cardiovascular disease is miR-155. One study showed the increased levels of this miRNA in aortic samples of *ApoE*^−/−^ mice, which develop atherosclerosis spontaneously. miR-155 and *ApoE* simultaneous knockout results in mice showing a decrease in atherosclerosis development but with increased obesity and aggravated NAFLD, suggesting that an increase of this miRNA is only detrimental in vascular tissue. In contrast, miR-155 upregulation promotes aortic endothelial cell activation by the induction of the expression of intercellular adhesion molecule 1 (87). According to this finding, a study carried out in 400 individuals showed elevated levels of miR-155 in the serum of patients with coronary heart disease and a positive correlation between the expression of this miRNA and the severity of the disease (88). miR-145 has also been pointed out as a miRNA whose expression is altered in CAD. In a study including 195 individuals, the miR-145 circulating levels were decreased in CAD patients, showing a progressive decrease as the disease aggravates (89). Interestingly, this miRNA has a role in attenuating T2DM and atherosclerosis. Bearing in mind that miR-145 is abundantly expressed in the vessel walls, its role in atherosclerosis may be protective since its target genes are osteoprotegerin and Kruppel-like factor 5, thus regulating the inflammatory response (90). In this regard, Lovren et al. described a decrease in the expression of miR-145 in the aorta of ApoE^−/−^ mice and the carotids of patients with atherosclerosis. It has previously been described that miR-145 promotes plaque stability by increasing the number of vascular smooth muscle cells, collagen content, and fibrous cap area as well as decreasing the number of macrophages and the necrotic area (91).

Indeed miR-145 and miR-155 have been mentioned many times with other miRNAs as candidates for diagnosis of CAD because of their expression patterns in the blood of CAD patients. The miR-145 and miR-155 levels were lower in the plasma of 67 patients, along with miR-126, miR-17, and miR-92a, whereas miR-133a and miR-208a were increased (92). This was later supported by a study that showed that the levels of miR-145, miR-155, and let-7 family member let-7c are lower when comparing CAD patients and healthy subjects (93). Although miR-145 and miR-155 expression seems to be generally altered in CVD, the involvement of the latter in this process remains unclear because of the inconsistencies found regarding its expression levels.

### Other miRNAs

Plenty of other miRNAs have been described as potential biomarkers for CAD and other CVDs. For instance, an analysis of serum samples from non-atherosclerotic and atherosclerotic patients revealed that miR-454, miR-744, miR-372, and miR-212 are differentially expressed between these two groups, although only miR-212 showed a discrimination power when combined with other atherosclerosis risk factors (94). In contrast, a study performed in 50 CAD patients showed the elevation of miR-93a-5p, miR-15a-5p, and miR-16-5p levels and the decrease of miR-146a-5p level in plasma (95). Parahuleva et al. predicted the impact of miR-92a on atherosclerosis and pointed out many target genes, such as NADPH oxidase and its impact on vascular oxidative stress, TNF receptor associated factor 3, which modulates CD40 signaling in atherogenesis, as well as protein kinase C ε and its implication in inflammation and smooth muscle cell dysfunction (96–99). Indeed the atherosclerotic plaque itself shows altered miRNA expression: higher levels of miR-21, miR-92a, and miR-99a and a lower expression of miR-1, miR-22, and let-7f were found in the atherosclerotic plaques of 12 patients, opening a new path for the search of putative biomarkers in cardiovascular alterations associated with NAFLD (96).

## miRNA as Biomarkers in the Diagnosis of Patients With NAFLD/NASH

Owing to the critical role of miRNAs as important epigenetic factors and their regulation of lipid and cholesterol biosynthesis in hepatocytes, these molecules are recently considered to be powerful biomarkers to diagnose hepatic diseases. miRNA involvement likewise ought to be strongly evaluated as follow-up and therapeutic tools in order to urgently find non-invasive strategies capable of replacing the only reliable method to differentiate between conditions, liver biopsy (100). Consequently, a key requirement to prove miRNA sensitivity and specificity would be large clinical cohorts suffering from NAFLD or related diseases. Despite the current drawbacks that miRNA-based technology presents, another useful feature that miRNAs possess is being targets for mimics and antisense oligonucleotides in case of undergoing an alteration in a specific disease (101). In addition, miRNAs are molecules that circulate into the bloodstream, where their expression levels are stable and easily quantitated, adding another reason to consider them as suitable clinical biomarkers. Specifically, the more proper option would be performing combined multiple miRNA analyses, as miRNA serum expression is also subject to the presence of non-liver-related disorders (102). In this sense, different authors have proposed miRNA panels for a high diagnostic accuracy of NAFLD. Whereas, Tan et al. proposed miR-122-5p, miR-1290, miR-27b-3p, and miR-192-5p for the diagnosis of NAFLD, Pirola et al. found that miR-192-5p or miR-122, miR-192, miR-19a, miR125, and miR-375 could also be an alternative panel (29, 103). However, other authors have suggested the combination of RNA panels with classical biomarkers for the prediction of NASH, specifically miR-122, miR-192, and miR-21 together with ALT and cytokeratin-18-Asp396 (52).

Furthermore, miRNAs could also be useful tools to assess NAFLD progression. Indeed miR-192 and miR-375 are associated with NAFLD activity score and classical biomarkers such as cytokeratin-18 (103). miR-122 in serum may likewise be a better predictor of NAFLD severity than classical biomarkers, such as ALT and cytokeratin-18 (29).

For all these reasons, miRNAs are becoming one of the few opportunities to advance and help, together with other classical biomarkers, in the diagnosis and prognosis of NAFLD.

## Conclusions

In the last decade, an increasing number of papers have highlighted the great diversity of functions that miRNAs have in the pathophysiology of metabolic diseases such as NAFLD, T2DM, and CVD. Indeed apart from the high amount of human genes regulated by miRNAs (104), miRNAs themselves are aberrantly expressed in many human diseases (105–108). In spite of the number of miRNAs identified in mouse and humans and the fact that some of them have been involved in hepatic pathogenesis, data regarding their specific roles are still scarce, and more efforts are needed to unravel the precise mechanisms. This task is quite challenging since a single miRNA can regulate hundreds of potential mRNA targets and different miRNAs can act on a particular mRNA target in a synergic or antagonic manner.

In this sense, manipulating miRNA expression whose targets are well-defined could represent a stage-specific therapy for NAFLD patients. For instance, both miR-122 and miR-21 are involved in the regulation of genes related to lipid metabolism, thus positioning them as candidates for the treatment of the earliest NAFLD stages, characterized by liver steatosis. Other miRNAs such as miR-34a, which regulates targets that participate in oxidative stress and inflammation, might be better suited for the treatment of individuals that have already progressed toward NASH. Moreover, since the development of NAFLD is tightly associated with the onset of T2DM and CVDs and all of them are promoted by subjacent systemic inflammation and metabolic alterations, it would be reasonable to expect that these diseases share alterations in the expression of some miRNAs. Therefore, the CVD-related miR-92a and miR-155 or the T2DM-associated let-7 and miR-200 could suppose novel and yet undiscovered targets for NAFLD treatment, with the potential to alleviate not only hepatic alterations but also other metabolic syndrome manifestations.

Finally, despite the recent advances that have been made about circulating miRNAs as potential biomarkers for NAFLD and the related diseases T2DM and CVD, in our opinion, the real challenge is to find more accurate correlations between the circulating levels of these miRNAs and the disease stage. In this way, these molecules could serve as powerful non-invasive diagnostic and prognostic tools that nowadays are a must for clinicians to avoid liver biopsy. Unfortunately, by now, much effort is still needed for this important challenge.

## Author Contributions

All authors listed have made a substantial, direct and intellectual contribution to the work, and approved it for publication.

## Conflict of Interest

The authors declare that the research was conducted in the absence of any commercial or financial relationships that could be construed as a potential conflict of interest.
